# Fatigue Testing of Different Treatment Options for a Bone Cavity: A Randomized, In Vitro, Biomechanical Cadaver Study in the Proximal Tibia of Goats

**DOI:** 10.7759/cureus.89874

**Published:** 2025-08-12

**Authors:** Gipson Samuel, Phiroj Rain, Anand Kumar, Pasupathy Palaniappan, Deepak Barathi, Shylvia Christy

**Affiliations:** 1 Orthopaedics, Jawaharlal Institute of Postgraduate Medical Education and Research, Puducherry, IND; 2 Orthopaedics, JR Medical College and Hospital, Tindivanam, IND; 3 Radiodiagnosis, Jawaharlal Institute of Postgraduate Medical Education and Research, Puducherry, IND; 4 Obstetrics and Gynaecology, Jawaharlal Institute of Postgraduate Medical Education and Research, Puducherry, IND

**Keywords:** bending stress, bone cavity, bone cement, bone cement supplemented with nail, bone cement supplemented with plate, fatigue failure, giant cell tumor of bone, goat cadaver, intralesional curettage, sn curves

## Abstract

Background

Giant cell tumor of the bone is mostly managed with extended intralesional curettage. A bone cavity after curettage is mostly managed with bone cement or bone cement supplemented with nail or plate. This study aimed to determine the treatment option for a bone cavity with maximum time to fatigue failure under sinusoidal reverse bending stress. The results of this study will help determine the standard treatment for a bone cavity.

Methodology

In this study, bone cavities managed with different treatment options in the proximal tibia of goat cadavers were subjected to sinusoidal reverse bending stress until fatigue failure. The time taken to fatigue failure was used to plot the SN curves for the different treatment options, and the SN curves were analyzed for significance.

Results

Of the 317 goat specimens, 60 satisfied the inclusion criteria. The mean time to fatigue failure was maximum for the bone cavity treated with bone cement supplemented with a plate. The mean time to fatigue failure (seconds) for bone cement supplemented with a plate was 924 ± 74.4, 606.5 ± 102.8, and 271.2 ± 32.7 for cyclical bending loads of 1, 3, and 5 kg, respectively. The Mann-Whitney U test was used to detect a significant difference between the two independent treatment groups using bone cement supplemented with nail and bone cement supplemented with a plate. The test statistic Z was -1.99, and the p-value for a two-tailed hypothesis was 0.04.

Conclusions

Based on this in vitro biomechanical study on goat cadavers, we recommend treatment of the bone cavity after intralesional curettage with bone cement supplemented with a plate for maximum resistance to mechanical stress and early rehabilitation.

## Introduction

Giant cell tumor (GCT) of the bone is a benign, aggressive tumor [1]. It is one of the most common bone tumors in India [2]. GCT is mostly managed with extended intralesional curettage [3], which leads to the development of a bone cavity. A bone cavity is ideally managed with cancellous bone graft [4]. However, because of the non-availability of large volumes of cancellous bone graft, bone cavities are mostly managed with bone cement or bone cement supplemented with a nail or plate [5].

The durability of the different treatment options for a bone cavity is not known due to a lack of adequate clinical experiments and biomechanical tests [6]. The common treatment options for bone cavities do not undergo remodelling, such as cancellous bone grafts, and hence, are susceptible to cumulative mechanical stress or fatigue. Bone cement is more resistant to axial stress than tension stress [7]. The fatigue resistance of the different treatment options for a bone cavity to cyclical bending stress reflects the durability of the treatment options. A previous study measured fatigue resistance of solder joints using a machine designed to apply flexural fatigue loading to cantilevered test specimens. The specimens were rotated around their main axis using a motor and weights loaded to the cantilevered test specimen applied a sinusoidal reverse bending stress to the solder joints [8].

The macrostructure of goat bones is similar to human bones [9]. In this study, we aimed to determine the treatment option for bone cavities in the proximal tibia of goat cadavers with maximum time to fatigue failure under sinusoidal reverse bending stress. The null hypothesis for the study is that the fatigue resistance of the bone cavity treated with bone cement supplemented with a plate is not better than the fatigue resistance of other treatment options. The primary objectives of the study were to determine the time taken for fatigue failure and plot the SN (stress-number of cycles to fatigue failure) curves for the different treatment options of a bone cavity.

In this study, we chose the tibia of male goat cadavers with similar anatomical axes, metaphyseal diaphyseal junctional diameters, and maximum epiphyseal diameters. We created a bone cavity of similar volume in the proximal tibia of goat cadavers. We then randomized and subjected the bone cavity to the different treatment options. The bone cavities managed with the different treatment options were then subjected to sinusoidal reverse bending stress until fatigue failure. The time taken to fatigue failure was used to plot the SN curves for the different treatment options, and the SN curves were analyzed for significance. The results of this study will help determine the standard treatment for a bone cavity.

## Materials and methods

Study design

In this study, bone cavities were randomized to the different treatment options and subjected to biomechanical fatigue testing.

Description of intervention

The bone cavity was created in the anteromedial surface of the proximal tibia of male goat cadavers with a small saw blade (Conmed powered instruments system, HALL), leaving 5 mm of subchondral bone. The height of the bone cavity was 3 cm, and the volume of the bone cavity was approximately 5 mL. The bone cavity was then subjected to one of the following four different treatment options: (1) bone cement (Subiton Quirurgico RO); (2) bone cement supplemented with five holed stainless steel (SS) low profile one-third tubular plate of thickness 2 mm (Bombay Ortho) fixed to cement and bone with five 3.5 mm SS cortical screws (Bombay Ortho) of length ranging between 20 and 25 mm; (3) bone cement supplemented with three 2 mm SS K wires (Bombay Ortho) of length 5 cm inserted longitudinally into the bone cement one after another before setting of the bone cement; and (4) resection of the bone cavity followed by cemented intramedullary prosthesis with a stem length and diameter of 5 cm and 5 mm, respectively.

Study population

**Figure 1:**
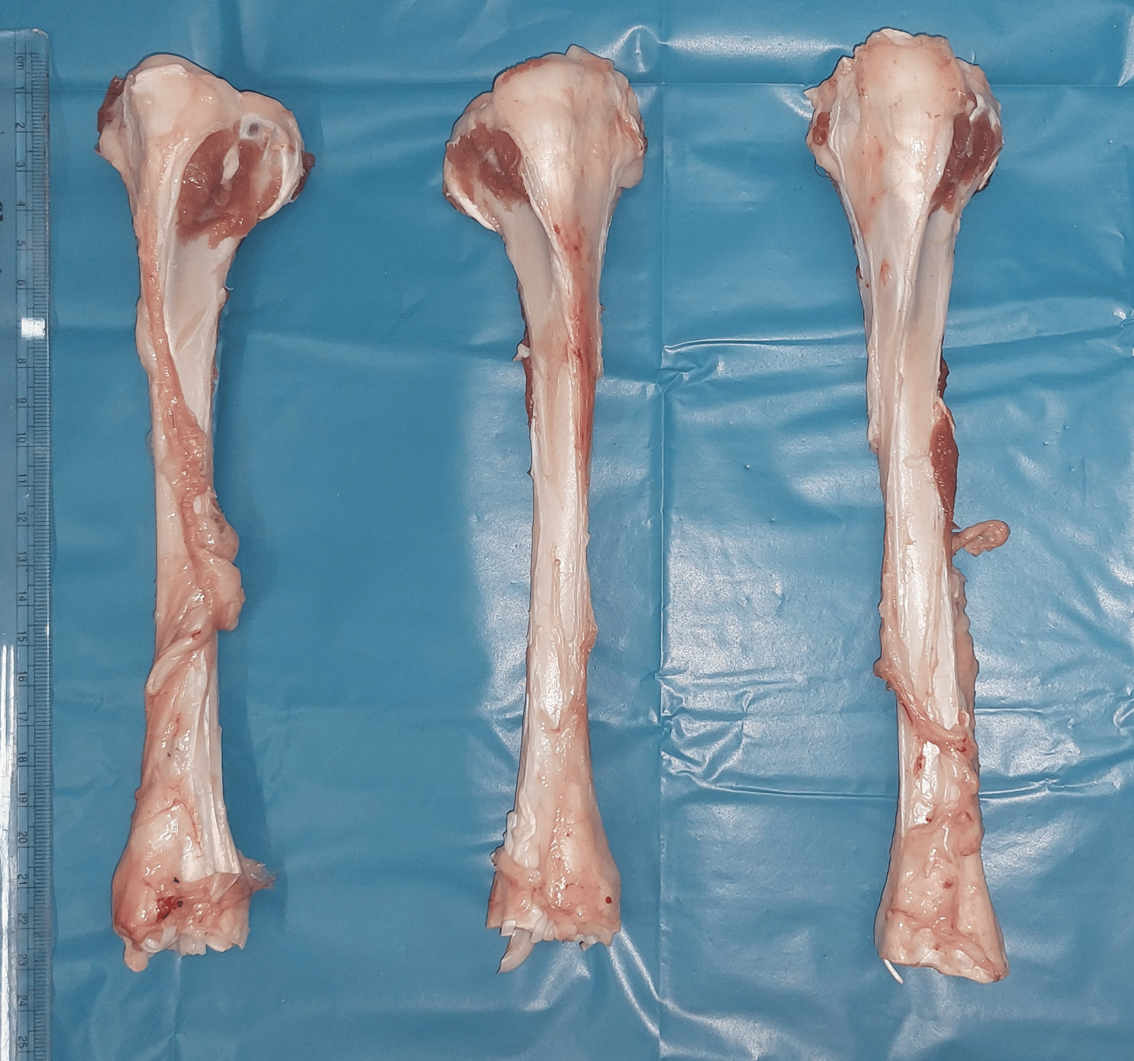
Fresh goat tibia specimens collected from a licensed abattoir.

**Figure 2:**
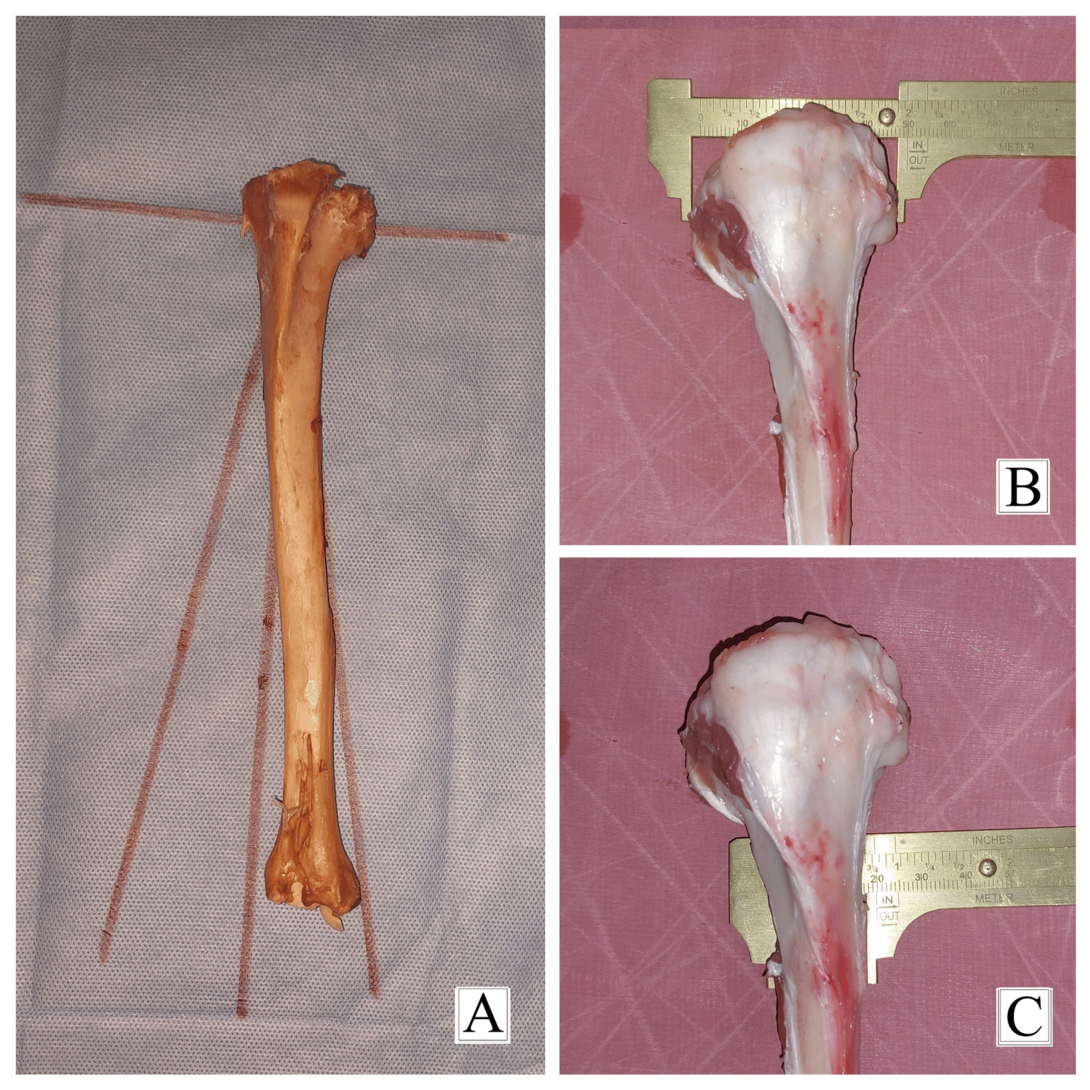
Specimens satisfying the inclusion criteria. (A) Goat tibia specimen with an anatomical axis of 80 degrees. (B) Goat tibia with a maximum epiphyseal diameter of 5 cm. (C) Goat tibia with a metaphyseal diaphyseal junctional diameter of 2 cm.

In this study, goats sacrificed within 24 hours for the purpose of consumption were selected. The goats were not sacrificed for the study. The macrostructure of goat bones is similar to human bones (Figure 1) [9]. Of the available goat specimens, the tibia of male goats with an anatomical axis of 80 degrees, metaphyseal diaphyseal junctional diameter of around 2 cm (1.9-2.1 cm) at 8 cm from the surface of the medial tibial plateau, and a maximum epiphyseal diameter of around 5 cm (4.9-5.1 cm) were included in the study (Figure 2). This study was conducted between August 2021 and August 2022 in a dedicated minor operation theatre in the Department of Orthopaedics.

Operational definitions

The independent variables were the weights loaded on the test specimen to create bending stress, i.e., 1, 3, and 5 kg. The dependent variable (continuous variable) was the time to fatigue failure in seconds.

Sampling technique

The goat specimens were sampled by convenience sampling, a non-probability type of sampling technique. The goat specimens were randomized into the different treatment options by simple randomization.

Sample size

The major study variable was the time to fatigue failure in seconds. The time to fatigue failure was summarized as mean ± standard deviation (SD). Sample size was calculated in Statulator.com [10] using the sample size calculator for comparing two independent means. We assumed a true difference in means between the treatment options using bone cement with a nail and bone cement with a plate of 300 seconds. We calculated the pooled standard deviation as 200 seconds by assuming that the time to fatigue failure would range between 0 and 800 seconds. Using the sample size calculator for comparing two independent means, the study required a sample size of 12 for each group to achieve a power of 80% and a significance level of 5% for declaring that the treatment option using bone cement with a plate is superior to the treatment option using bone cement with a nail at 100 seconds margin of superiority. In other words, if we select a random sample of 12 from each group and determine the difference in the two means as 300 seconds and the pooled SD as 200 seconds, we will have 80% power to declare that the mean of the treatment option using bone cement with a plate is at least 100 seconds higher than the mean of the treatment option using bone cement with a nail.

Study procedure

**Figure 3:**
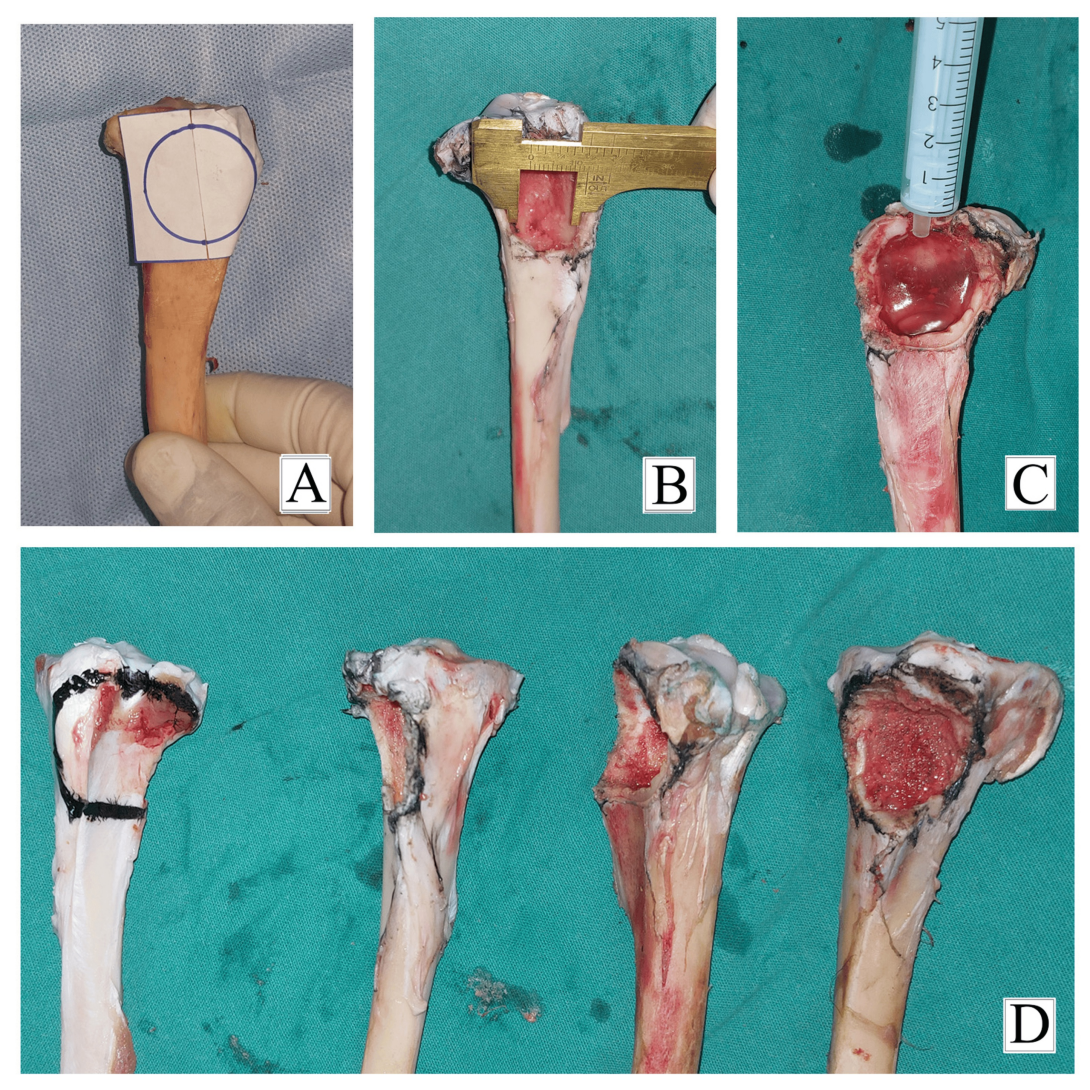
Preparation of bone cavity in the proximal tibia of goat specimens. (A) Bone window of diameter 2 cm, 5 mm from the joint line. (B) Bone window of diameter 2 cm as measured with the vernier calliper. (C) Bone cavity with a volume of 5 mL. (D) Goat specimens after preparation of the bone cavity.

**Figure 4:**
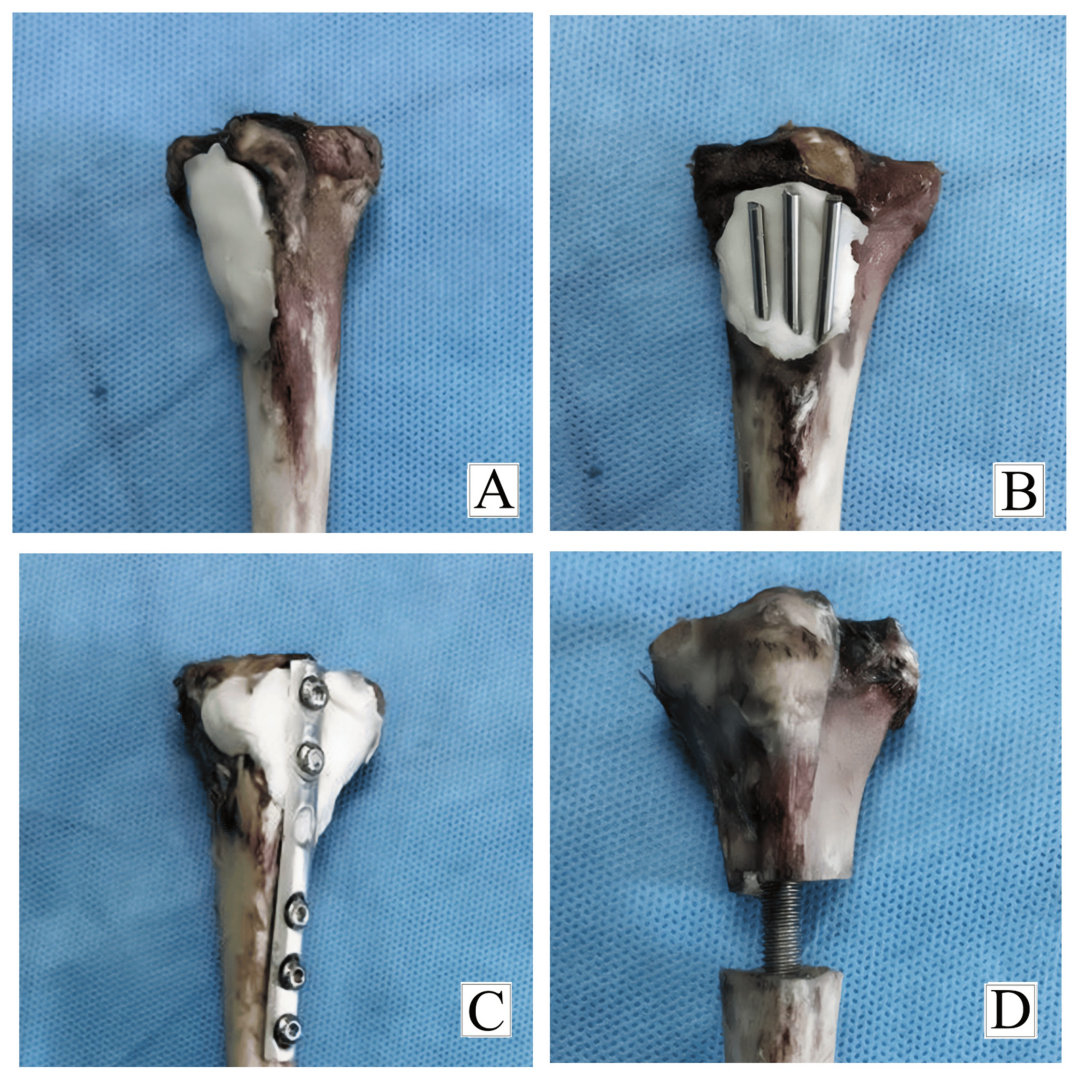
Bone cavity after treatment. (A) Bone cavity after treatment with bone cement. (B) Bone cavity after treatment with bone cement supplemented with nail. (C) Bone cavity after treatment with bone cement supplemented with plate. (D) Osteotomy at the metaphyseal diaphyseal junction stabilized with an intramedullary prosthesis.

**Figure 5:**
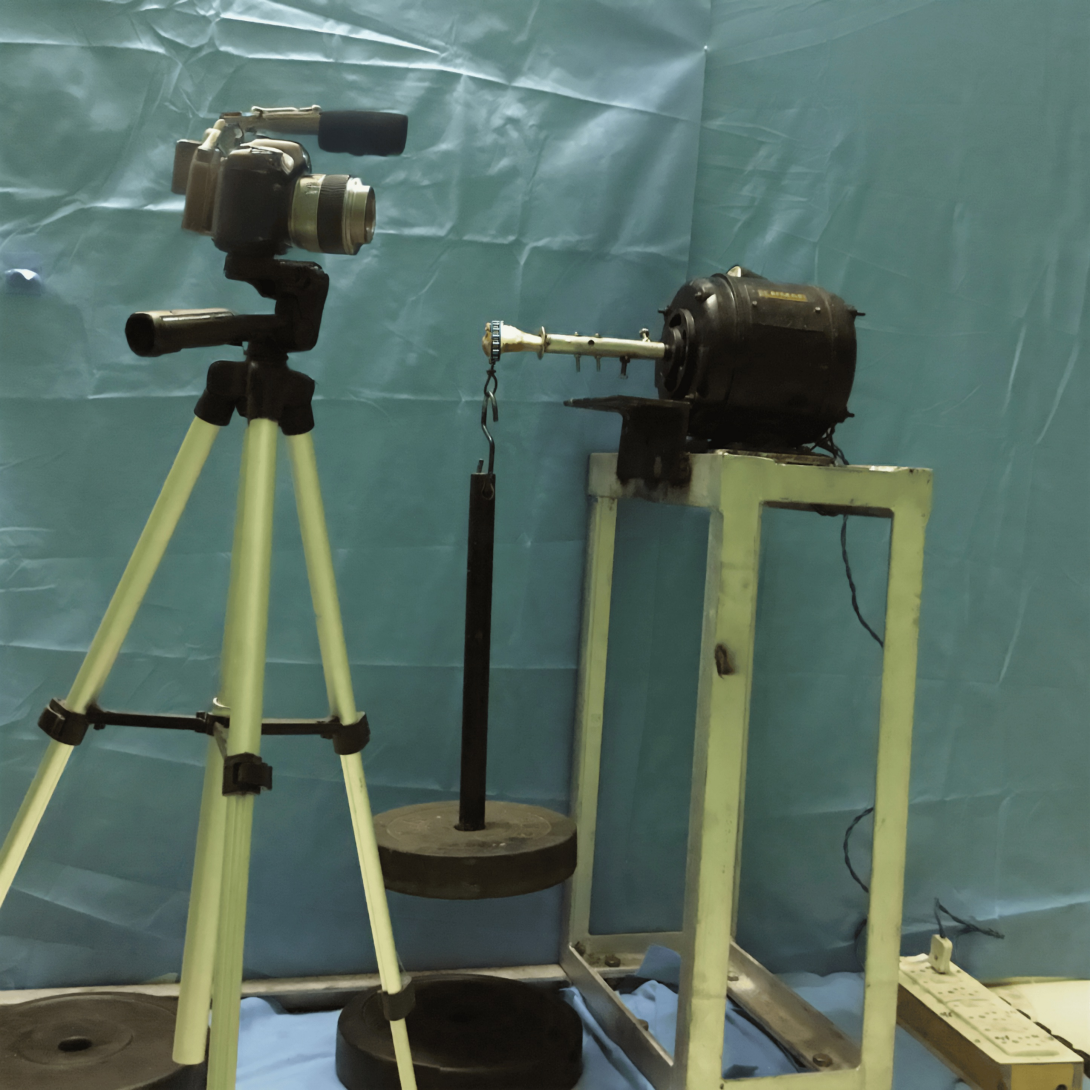
The goat specimen cantilevered for applying sinusoidal reverse bending stress to measure fatigue resistance.

Goat specimens were collected from government-authorized slaughterhouses. The goat tibias included in the study were prepared by removing the soft tissues around the proximal end of the tibia. A bone window was then created using a mini saw (Conmed Power System, HALL) on the anteromedial surface of the proximal tibia, such that the diameter of the window of the bone cavity was 2 cm and the margin of the bone window was 5 mm from the joint surface. The bone window was then deepened using a curette and burr to form a bone cavity of around 5 mL (Figure 3). The bone cavity was prepared, leaving behind 5 mm of subchondral bone toward the knee joint. After the creation of the bone cavity, each bone cavity was assigned a treatment by simple block randomization. The different treatment options were (1) bone cement, (2) bone cement supplemented with nail, (3) bone cement supplemented with plate, and (4) intramedullary prosthesis (Figure 4). The bone cavities treated with the different treatment options were then subjected to sinusoidal reverse bending stress to measure fatigue resistance. The proximal end of the treated bone cavity was secured to a one-horsepower motor (Kumar Motor) with the help of bolts and bone cement and rotated around its main axis at 1,800 rotations per second. The distal end of the treated bone cavity was then fixed to a bearing which was holding the weights that applied flexural fatigue loading (Figure 5). The experiment to measure the fatigue resistance was repeated four times with 1 kg, four times with 3 kg, and four times with 5 kg to make up a sample size of 12 per treatment group.

The experiment was recorded with a video camera (Panasonic GX85) at 60 frames/second to calculate the time to fatigue failure. Fatigue failure was defined as the time at which the treated bone cavity bent more than 45 degrees to the long axis of the bone, leading to the disruption of the experiment. The time to fatigue failure in seconds for each experiment was entered into a Microsoft Excel workbook (version 2018) (Microsoft Corp., Redmond, WA, USA).

Statistical analysis

Data were entered in a Microsoft Excel workbook (version 2018). Statistical analysis was performed using SPSS Statistics version 26.0 (IBM Corp., Armonk, NY, USA). The time to fatigue failure for the different treatment options was the continuous variable expressed in seconds and summarized as mean ± SD. SN curves were plotted for each treatment option of the bone cavity, with the weight applying bending stress in the X axis and the time to fatigue failure in seconds in the Y axis. The SN curves were then analyzed for significance with the Kolmogorov-Smirnov (KS) test and the Mann-Whitney U test [11,12].

We adhered to good laboratory practice when handling animal tissues. We considered all animal tissues as potentially infectious and handled them with proper aseptic precautions. All instruments and equipment were disinfected in an autoclave after use. The study protocol and proforma were approved by the institute’s scientific committee before the beginning of the study. This study was exempted from human ethics review as it was an in vitro biomechanical study involving goat cadavers obtained from animal sacrifice for consumption and did not involve human participants. As these animals were not sacrificed for the study, this study was exempted by the institute’s animal research committee. This study was funded by the institute’s intramural fund. CONSORT reporting guidelines were used for this randomized biomechanical study [13].

## Results

**Figure 6:**
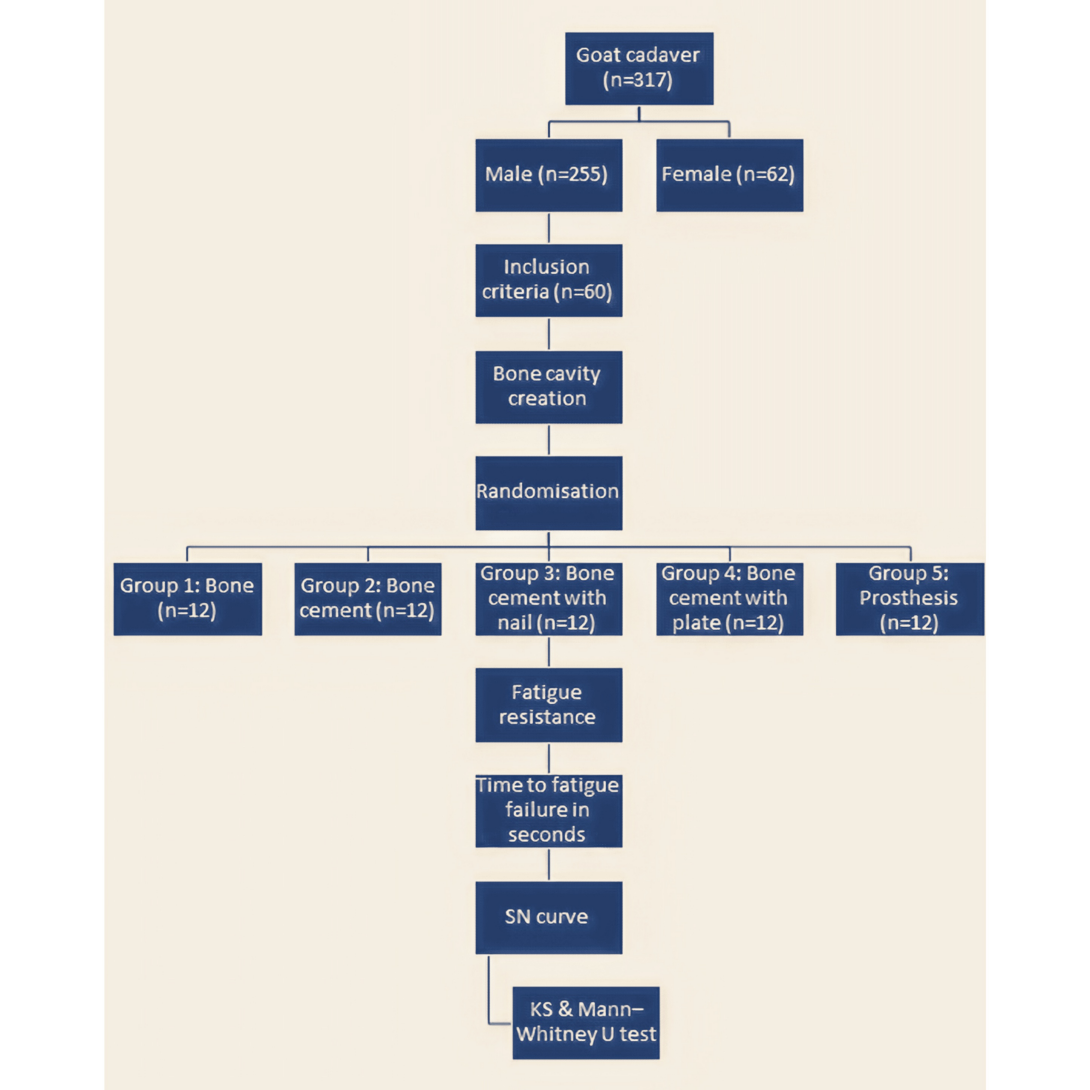
Flowchart of the test specimens.

In this study, male goat tibias of similar dimensions were chosen and subjected to the creation of bone cavities. The bone cavities were then randomized into different treatment options to measure the fatigue resistance of the different treatment options for the bone cavities. The primary outcome variable in this study was the time to fatigue failure in seconds, and the independent variable was the bending load applied to the test specimens in kg. Figure 6 shows the study flowchart.

**Figure 7:**
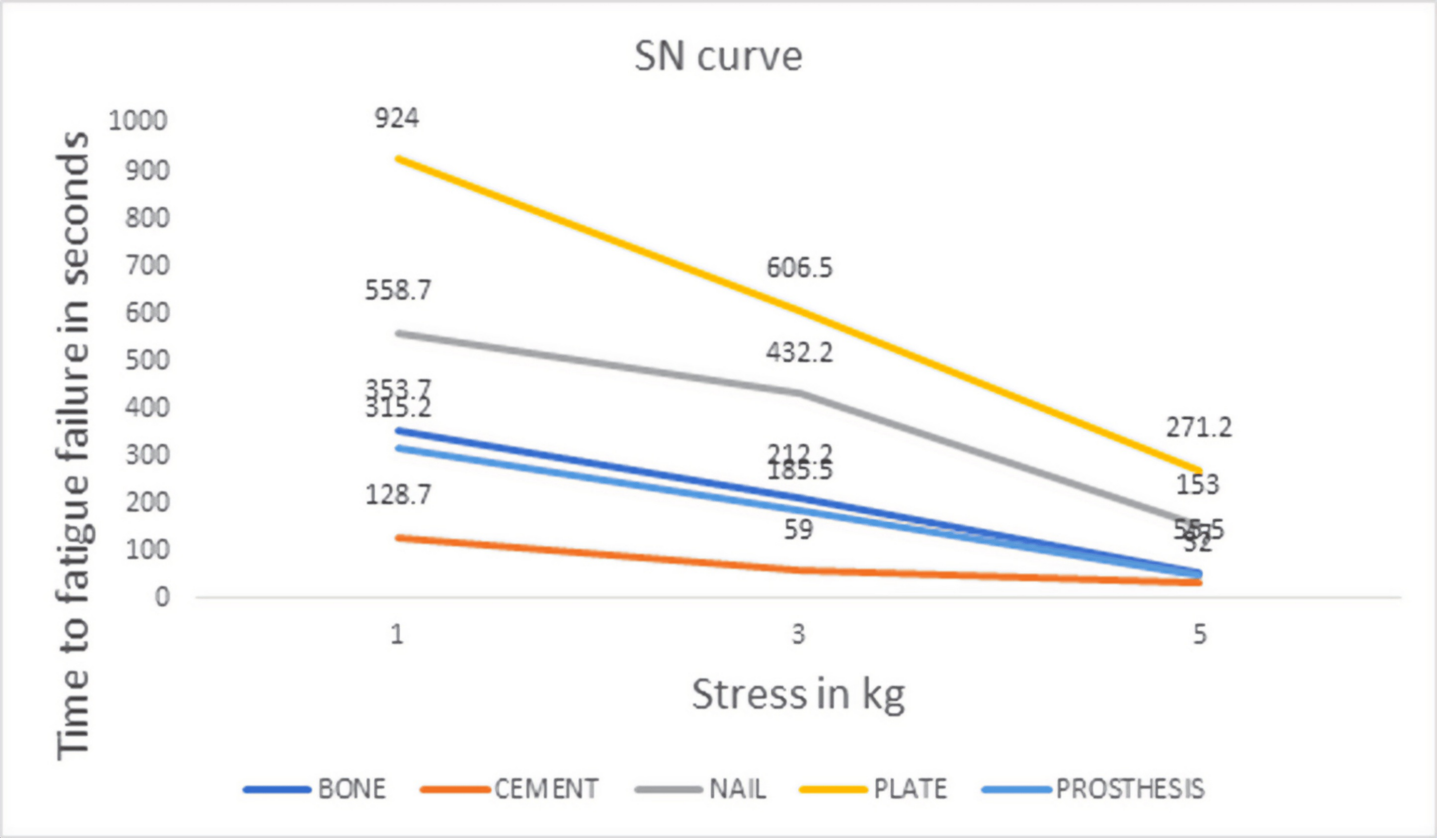
SN curve plotted with the mean time to fatigue failure.

**Figure 8:**
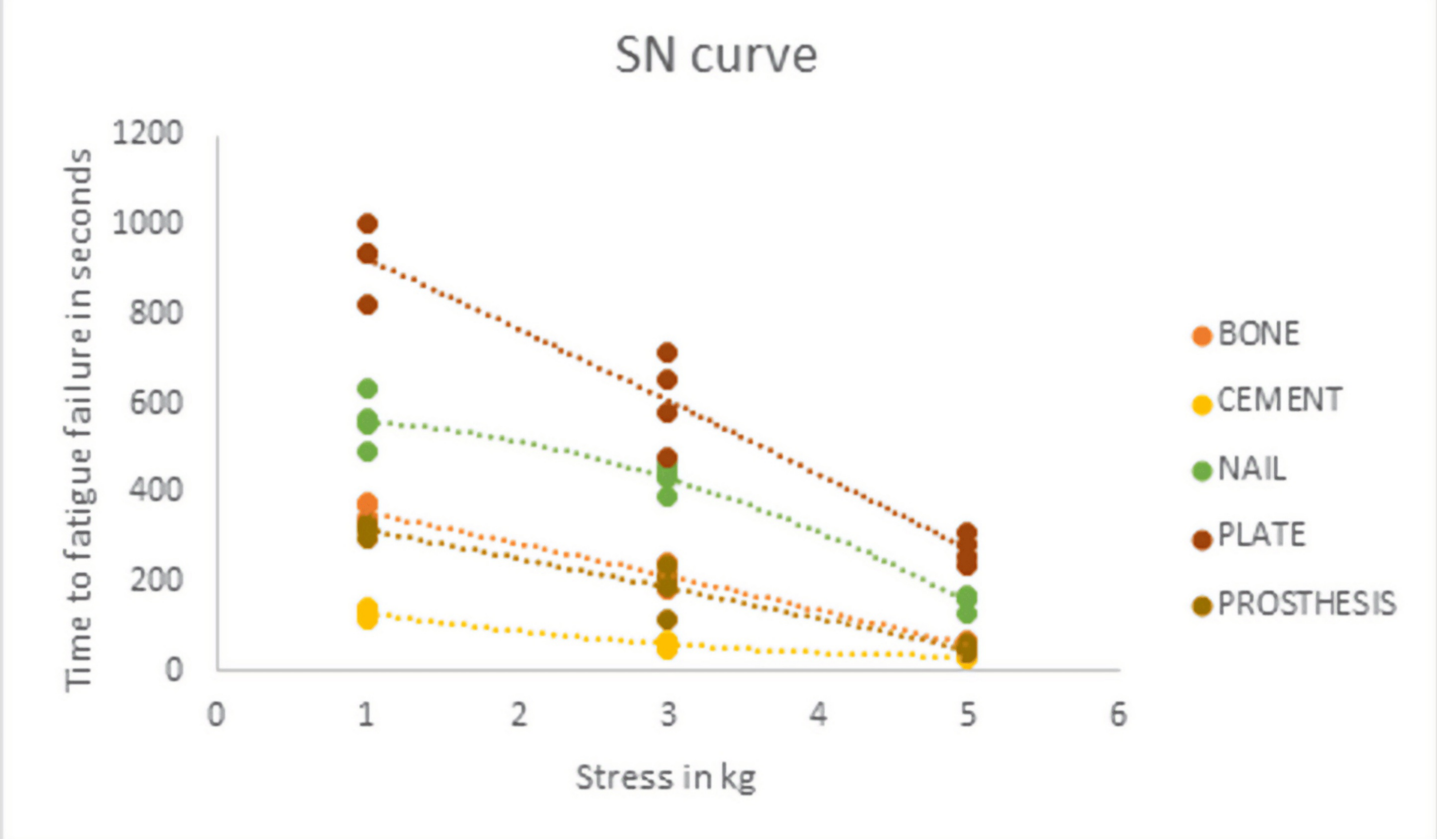
Scatter diagram illustrating the time to fatigue failure.

Of the 317 goat specimens examined in the animal slaughterhouses, 255 were male and 62 were female. Of the 255 male goat specimens, 60 satisfied the inclusion criteria. The mean metaphyseal diaphyseal junctional diameter of the proximal end of male goat tibia included in the study, 8 cm from the surface of the medial tibial plateau, was 1.98 ± 0.07 cm. The mean maximum epiphyseal diameter of the proximal end of the male goat tibia included in the study was 5.01 ± 0.07 cm. The thickness of the cortex of the goat tibia was measured to be approximately 2 mm at the distal extent of the bone window in the anteromedial surface. Table 1 shows the mean time to fatigue failure for the different treatment options of a bone cavity. The mean time to fatigue failure was maximum for the bone cavity treated with bone cement supplemented with a plate. The mean time to fatigue failure (seconds) for bone cement supplemented with a plate was 924 ± 74.4, 606.5 ± 102.8, and 271.2 ± 32.7 for cyclical bending loads of 1, 3, and 5 kg, respectively. The mean time to fatigue failure was minimum for the bone cavity treated with bone cement alone. The mean time to fatigue failure (seconds) for bone cement was 128.7 ± 9.7, 59 ± 8.6, and 32 ± 4.5 for cyclical bending loads of 1, 3, and 5 kg, respectively. Figure 7 and Figure 8 show the SN curves plotted with the mean time to fatigue failure of the different treatment options and a scatter diagram of all the individual time to fatigue failure measurements, respectively.

**Table 1:** Mean time to fatigue failure.

Group	Tension load (kg)	Mean time to fatigue failure (seconds)
Normal bone	1	353.7 ± 24.9
	3	212.2 ± 26.3
	5	55.5 ± 8.4
Bone cement	1	128.7 ± 9.7
	3	59 ± 8.6
	5	32 ± 4.5
Bone cement supplemented with a nail	1	558.7 ± 58.3
	3	432.2 ± 28.5
	5	153 ± 18.8
Bone cement supplemented with a plate	1	924 ± 74.4
	3	606.5 ± 102.8
	5	271.2 ± 32.7
Intramedullary prosthesis	1	315.2 ± 12.7
	3	185.5 ± 49.6
	5	47 ± 10.7

**Figure 9:**
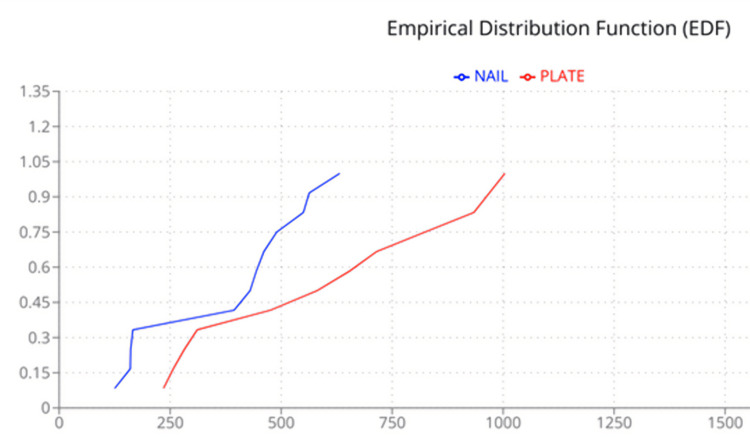
Empirical distribution function plot for bone cavities treated with bone cement supplemented with a nail and bone cement supplemented with a plate.

The SN curves showed the fatigue resistance measured as the time to fatigue failure for the treatment options using bone cement supplemented with a plate and bone cement supplemented with a nail to be superior to the other treatment options for the bone cavity. The two-sample KS test was used to detect differences in the shape of the two distributions in the SN curve. Figure 9 shows the empirical distribution function (EDF) plot obtained from the time to fatigue failure of the treatment done with bone cement supplemented with a nail and plate. The KS statistic D was 0.5, and the p-value was 0.09. The Mann-Whitney U test was used to detect a significant difference between the two independent treatment groups using bone cement supplemented with a nail and bone cement supplemented with a plate. The Mann-Whitney test statistic U was 37. The expected value of U was 72. The standard error of U was 17.3. The test statistic Z was -1.99, and the p-value for a two-tailed hypothesis was 0.04.

## Discussion

This study was designed to determine the treatment option for a bone cavity with maximum resistance to mechanical stress. Goat bones are similar to human bones [9], and the resistance to cyclical bending load resulting in fatigue failure will help us determine the treatment with maximum resistance to stress. The mean time to fatigue failure was higher for the bone cavity treated with bone cement supplemented with a plate and nail when compared to the other treatment options. The SN curves also showed that the time to fatigue failure for the treatment options using bone cement supplemented with a plate and nail was superior to the other treatment options. The KS test did not find a significant difference in the treatment with bone cement supplemented with a nail or plate, with a p-value of 0.09. On the other hand, the Mann-Whitney U test, a non-parametric test sensitive to detecting differences in the median value, showed a significant difference between the treatment options using bone cement supplemented with a nail and plate, with a p-value of 0.04.

Treatment of the bone cavity with bone cement alone was very weak because the cement fractures easily on application of tension stress [7]. Treatment of a bone cavity with resection and cemented intramedullary prosthesis has similar strength to normal bone, as the point of fatigue failure is at the tip of the prosthesis. Treatment of the bone cavity with bone cement supplemented with a nail failed because the volume of cement inside the bone cavity was interrupted with multiple nails, decreasing the diameter of cement at the point of maximum stress. Treatment of the bone cavity with bone cement supplemented with a plate was resistant to tension stress, mainly because of the plate. Though the cement fractured early, the plate resisted tension stress to some extent before failure. It was also found that the plate did not bond with the cement, as it was applied to the surface of the cement. The treatment of the bone cavity with bone cement supplemented with a nail and plate failed due to cement fracture and bending of the nail and plate. The plate did not fail due to screw pullout.

Asavamongkolkul et al., Murray et al., Randall et al., Weiner et al., and Toy et al. compared the treatment of bone cavity with bone cement and bone cement supplemented with a nail [14-18]. They concluded that there was no significant difference in the treatment groups biomechanically. Uglialoro et al. compared the treatment of bone cavity with bone cement supplemented with a nail and bone cement supplemented with a locking plate in an in vitro study of 30 tibias [19]. They concluded that locking plates are stiffer and more resistant to stress. In all the above studies, compression stress was used for biomechanical testing. Bone cement is very resistant to compressive stress when compared to tension stress [7]. Hence, biomechanical testing under cyclical tension stress will give a more appropriate result for clinical decision-making. Ahmadi et al. compared the treatment of bone cavity with bone cement supplemented with a nail, bone cement supplemented with a locking plate, and bone cement supplemented with a non-locking plate [20]. They subjected 15 synthetic femurs to mechanical tests for axial, sagittal, coronal, torsional stiffness, and torsional strength. They concluded that nails performed better than plates.

This study aimed to address a lacuna and a common controversy in the current literature in the management of bone cavity [6]. The study evaluated the strength of the different treatment options for the bone cavity with cyclical bending stress. Cyclical bending stress leading to fatigue failure is more relevant clinically as bone cement is very strong in compression and very weak in tension [7]. This study showed a significant difference in favor of the treatment of bone cavities with bone cement supplemented with a plate compared to the previous study by Ahmadi et al. [20]. This study was a prospective, randomized, in vitro, biomechanical study with strict inclusion criteria and proper standardization of procedure to minimize selection bias, instrument bias, performance bias, and possible confounders. There are no conflicts of interest. The macrostructure of goat bones is similar to human bones [9]. The age of the goat bones from sacrifice was less than 24 hours, compared to experiments on human cadaver bones, where it is likely to be longer. The sample size was adequate. The mode of failure was at the expected normal diaphyseal cortical bone-bone cement junction, and all failures were due to cement fracture and bending of the implant used. There were no screw pullouts or pullouts of the test specimen from the equipment. The results of the study are comparable with similar studies. Though the macrostructure of goat bones is similar to human bones, the microstructure, bone composition, and bone remodelling of human bones are more similar to pig bones in comparison to goat bones [9]. Fatigue fracture of normal bone and the bone treated with a prosthesis occurred in normal bone. The normal bone can remodel, so the fatigue failure in clinical practice will not be the same as it is in this experiment.

Though this study was performed using goat cadavers, the study showed significant improvement in fatigue resistance for the treatment of bone cavities with bone cement supplemented with a plate, favoring such treatment in the clinical setting for better resistance to mechanical stress and early rehabilitation.

Limitations

The tibias of the goat cadavers in the slaughterhouses were of different dimensions. The proximal tibia of the goat specimens satisfying the inclusion criteria were alone included for the experiment to minimize selection bias. The bone window and the bone cavity were created with standard guides and cross-checked with vernier callipers (Mini Vernier callipers of 1mm resolution and 0.5mm accuracy, Lab world, India) to minimize performance bias. After the creation of the bone cavity, the thickness of the cortex in the proximal end of the bone window was also measured to minimize the confounding effect of the thickness of the cortex in the measurement of fatigue resistance. The preparation of bone, the creation of the bone cavity, the treatment of the bone cavity, and the experiment to measure fatigue resistance were done by a single orthopedic surgeon with assistance from a junior resident. The proximal and the distal ends of the treated bone cavity were fixed with the motor and the bearing holding the weights, respectively, with two bolts and nuts at right angles to each other, supplemented with bone cement. Bone cement supplementation helped prevent bolt cutout and pullout of the proximal or distal end of the treated bone cavity during the experiment.

## Conclusions

This in vitro biomechanical study demonstrated that among the various treatment options for managing a bone cavity after intralesional curettage, bone cement supplemented with a plate provides the highest resistance to fatigue failure under cyclical bending stress. The time to fatigue failure was significantly greater for this group compared to other modalities, including bone cement alone, bone cement with an intramedullary nail, and cemented intramedullary prosthesis. The results support the hypothesis that reinforcement with a plate enhances the structural integrity of the treated bone cavity, primarily by counteracting tension forces that plain cement is inherently weak against. These findings, derived from a rigorously standardized cadaveric goat model, have important implications for clinical practice and suggest that plate augmentation may provide better mechanical durability and potentially facilitate early rehabilitation in patients. Further validation using fresh frozen human cadaver models combined with finite element modelling is warranted to optimize and standardize this approach in clinical settings.
